# Supplementary material for: Sympathetic Burden Measured Through a Chest-Worn Sensor Correlates with Spatiotemporal Gait Performances and Global Cognition in Parkinson’s Disease
Source: Sensors (Basel). 2025 Sep 16;25(18):5756. doi: 10.3390/s25185756 (PMC12473974; doi:10.3390/s25185756)
Supplement: Supplementary file 1 [file sensors-25-05756-s001.zip › sensors-3823174-supplementary.pdf]

sensors

MDPI

Supplementary Materials S1. Scatterplot Diagrams of Clinical and Instrumental Findings

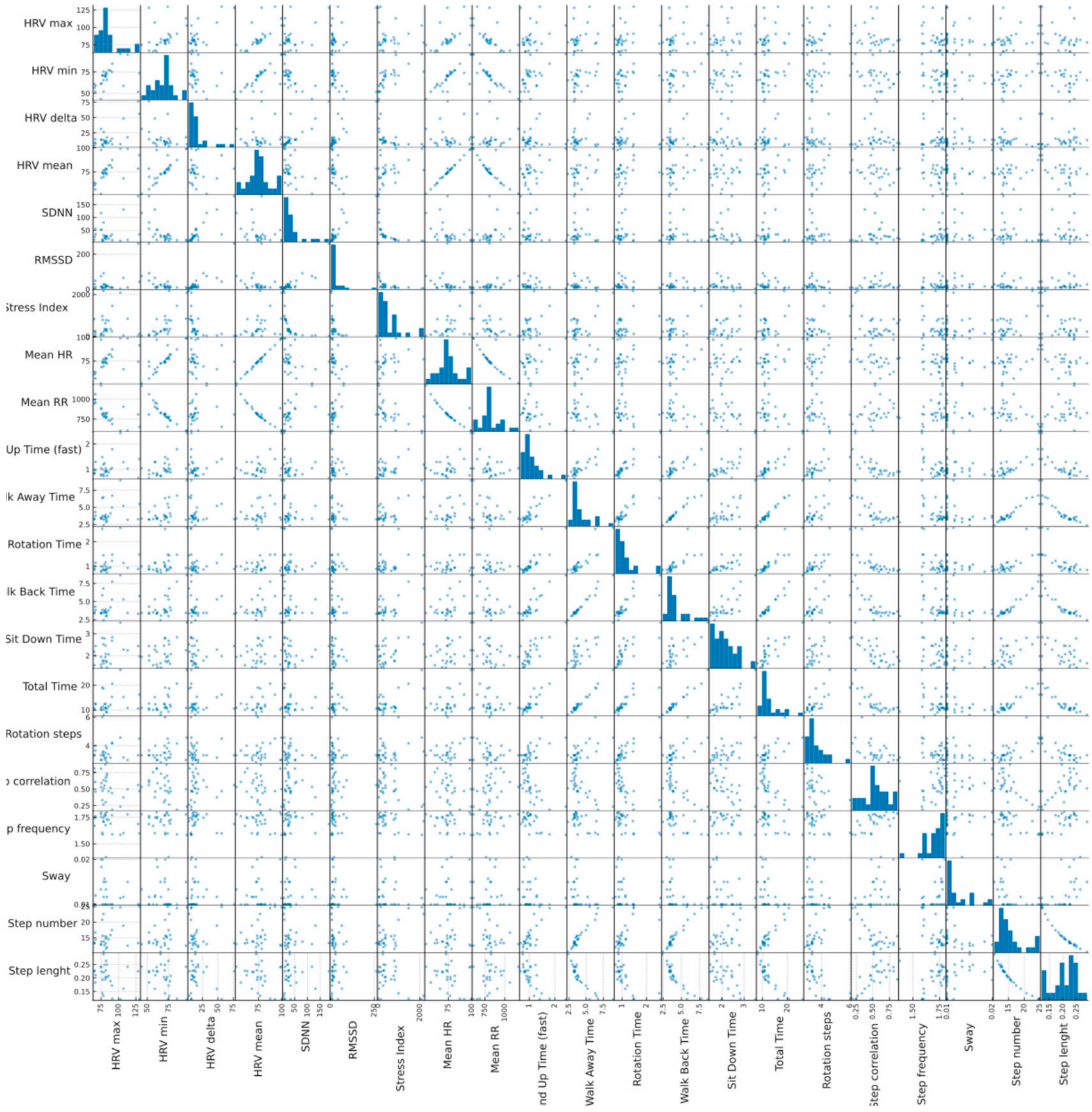

Figure S1. Scatterplot of correlations between HRV variables and spatiotemporal gait parameters during normal pace TUG.

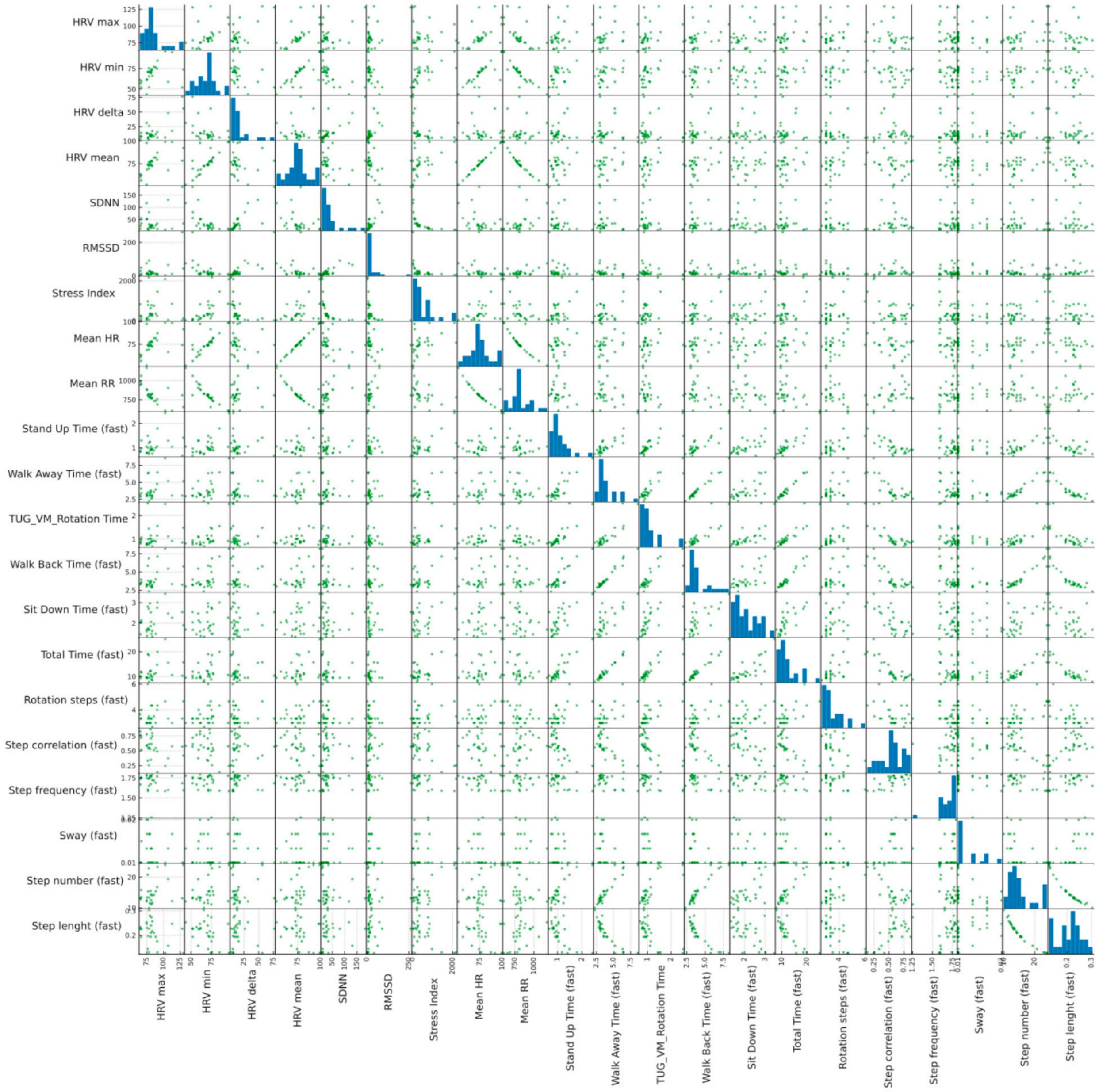

Figure S2. Scatterplot of correlations between HRV variables and spatiotemporal gait parameters during fast TUG.

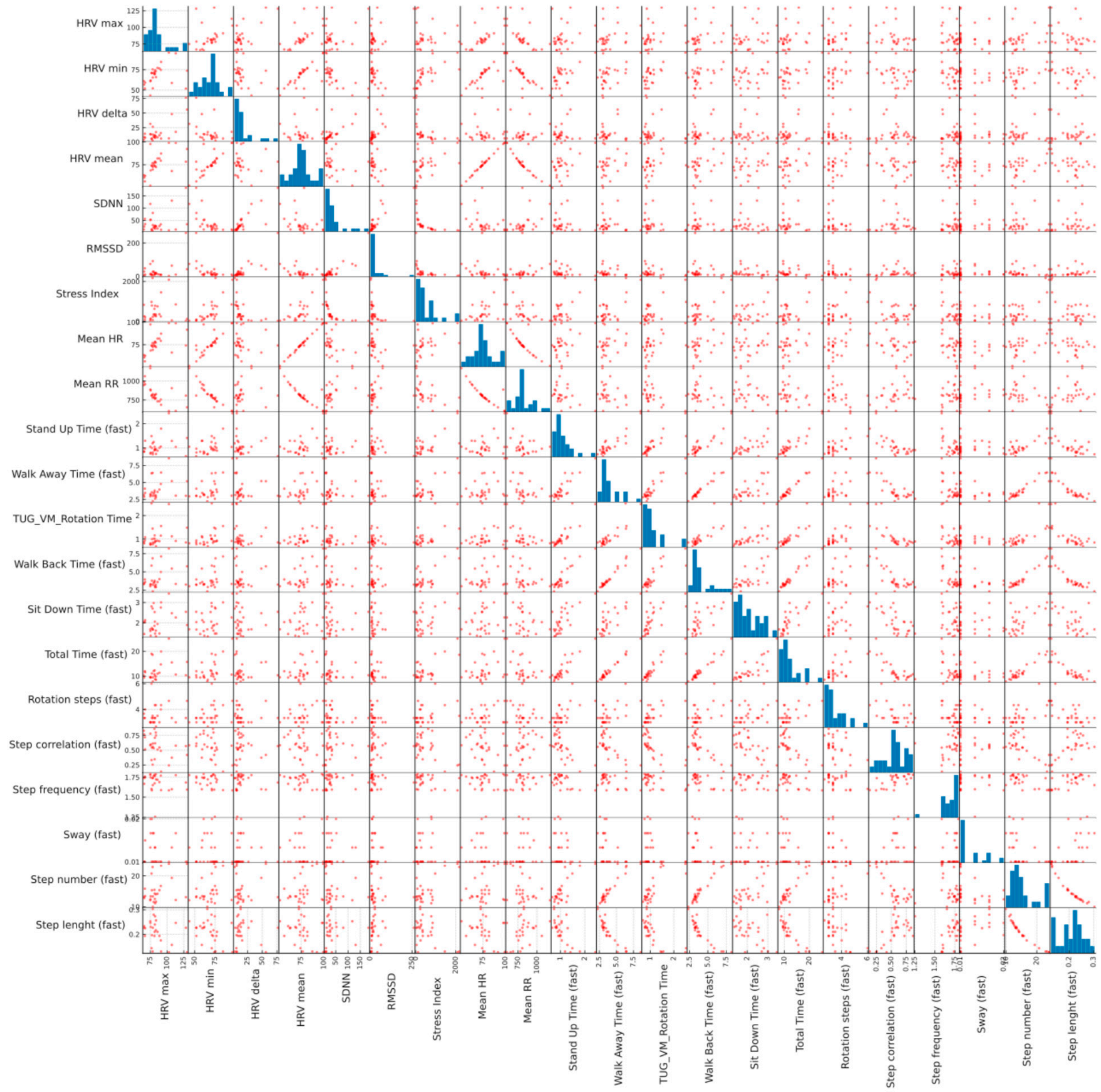

Figure S3. Scatterplot of correlations between clinical and demographical parameters and HRV variables.

Supplementary Materials S2. Statistical Test p-Values of Correlation Between Clinical Parameters and Sensor-Based Findings

Table S1. Statistical test p-values of correlations between HRV variables and spatiotemporal gait parameters during normal pace TUG.

Riga	HRV max	HRV min	HRV delta	HRV mean	SDNN	RMSSD	BSTRi	Mean HR	Mean RR	Stand Up Time	Walk Away Time	Rotation Time	Walk Back Time	Sit Down Time	Total Time	Rotation steps	Step correlatio n	Step frequency	Sway	Step number	Step length
HRV max	0,000	0,070	0,000	0,001	0,056	0,011	0,916	0,003	0,001	0,152	0,154	0,297	0,125	0,155	0,135	0,595	0,097	0,194	0,379	0,053	0,096
HRV min	0,070	0,000	0,049	0,000	0,000	0,005	0,000	0,000	0,000	0,002	0,009	0,019	0,006	0,021	0,005	0,190	0,024	0,167	0,803	0,022	0,047
HRV delta	0,000	0,049	0,000	0,902	0,000	0,000	0,012	0,852	0,840	0,500	0,698	0,564	0,718	0,840	0,674	0,686	0,957	0,774	0,492	0,780	0,821
HRV mean	0,001	0,000	0,902	0,000	0,126	0,416	0,031	0,000	0,000	0,028	0,044	0,100	0,041	0,138	0,042	0,487	0,209	0,355	0,522	0,060	0,139
SDNN	0,056	0,000	0,000	0,126	0,000	0,000	0,004	0,063	0,029	0,171	0,313	0,221	0,293	0,657	0,290	0,236	0,721	0,605	0,923	0,370	0,470
RMSSD	0,011	0,005	0,000	0,416	0,000	0,000	0,045	0,250	0,349	0,146	0,313	0,348	0,312	0,999	0,335	0,444	0,749	0,989	0,450	0,421	0,456
BSTRi	0,916	0,000	0,012	0,031	0,004	0,045	0,000	0,023	0,009	0,000	0,000	0,001	0,000	0,001	0,000	0,085	0,005	0,079	0,384	0,001	0,005
Mean HR	0,003	0,000	0,852	0,000	0,063	0,250	0,023	0,000	0,000	0,024	0,042	0,094	0,039	0,149	0,040	0,464	0,215	0,370	0,492	0,059	0,139
Mean RR	0,001	0,000	0,840	0,000	0,029	0,349	0,009	0,000	0,000	0,031	0,045	0,076	0,032	0,072	0,034	0,282	0,062	0,137	0,669	0,037	0,076
Stand Up Time	0,152	0,002	0,500	0,028	0,171	0,146	0,000	0,024	0,031	0,000	0,000	0,000	0,000	0,000	0,000	0,001	0,000	0,075	0,220	0,000	0,000
Walk Away Time	0,154	0,009	0,698	0,044	0,313	0,313	0,000	0,042	0,045	0,000	0,000	0,000	0,000	0,000	0,000	0,000	0,000	0,076	0,082	0,000	0,000
Rotation Time	0,297	0,019	0,564	0,100	0,221	0,348	0,001	0,094	0,076	0,000	0,000	0,000	0,000	0,000	0,000	0,000	0,000	0,048	0,160	0,000	0,000
Walk Back Time	0,125	0,006	0,718	0,041	0,293	0,312	0,000	0,039	0,032	0,000	0,000	0,000	0,000	0,000	0,000	0,000	0,000	0,068	0,072	0,000	0,000
Sit Down Time	0,155	0,021	0,840	0,138	0,657	0,999	0,001	0,149	0,072	0,000	0,000	0,000	0,000	0,000	0,000	0,001	0,000	0,360	0,101	0,001	0,002
Total Time	0,135	0,005	0,674	0,042	0,290	0,335	0,000	0,040	0,034	0,000	0,000	0,000	0,000	0,000	0,000	0,000	0,000	0,072	0,080	0,000	0,000
Rotation steps	0,595	0,190	0,686	0,487	0,236	0,444	0,085	0,464	0,282	0,001	0,000	0,000	0,000	0,001	0,000	0,000	0,005	0,161	0,085	0,000	0,000
Step correlation	0,097	0,024	0,957	0,209	0,721	0,749	0,005	0,215	0,062	0,000	0,000	0,000	0,000	0,000	0,000	0,005	0,000	0,032	0,312	0,000	0,000
Step frequency	0,194	0,167	0,774	0,355	0,605	0,989	0,079	0,370	0,137	0,075	0,076	0,048	0,068	0,360	0,072	0,161	0,032	0,000	0,679	0,068	0,152
Sway	0,379	0,803	0,492	0,522	0,923	0,450	0,384	0,492	0,669	0,220	0,082	0,160	0,072	0,101	0,080	0,085	0,312	0,679	0,000	0,036	0,016
Step number	0,053	0,022	0,780	0,060	0,370	0,421	0,001	0,059	0,037	0,000	0,000	0,000	0,000	0,001	0,000	0,000	0,000	0,068	0,036	0,000	0,000
Step length	0,096	0,047	0,821	0,139	0,470	0,456	0,005	0,139	0,076	0,000	0,000	0,000	0,000	0,002	0,000	0,000	0,000	0,152	0,016	0,000	0,000

Table S2. Statistical test p-values of correlations between HRV variables and spatiotemporal gait parameters during fast TUG.

	HRV max	HRV min	HRV delta	HRV mean	SDNN	RMSSD	BSTRi	Mean HR	Mean RR	Stand Up Time (fast)	Walk Away Time (fast)	Rotatiion time (fast)	Walk Back Time (fast)	Sit Down Time (fast)	Total Time (fast)	Rotation steps (fast)	Step correlation (fast)	Step frequency (fast)	Sway (fast)	Step number (fast)	Step length (fast)
HRV max	0,000	0,070	0,000	0,001	0,056	0,011	0,916	0,003	0,001	0,182	0,150	0,262	0,094	0,105	0,113	0,443	0,061	0,302	0,451	0,049	0,084
HRV min	0,070	0,000	0,049	0,000	0,000	0,005	0,000	0,000	0,000	0,002	0,007	0,008	0,006	0,024	0,005	0,091	0,026	0,223	0,805	0,025	0,057
HRV delta	0,000	0,049	0,000	0,902	0,000	0,000	0,012	0,852	0,840	0,478	0,682	0,496	0,829	0,985	0,719	0,667	0,790	0,884	0,572	0,740	0,735
HRV mean	0,001	0,000	0,902	0,000	0,126	0,416	0,031	0,000	0,000	0,047	0,041	0,067	0,037	0,111	0,037	0,298	0,116	0,425	0,613	0,062	0,138
SDNN	0,056	0,000	0,000	0,126	0,000	0,000	0,004	0,063	0,029	0,171	0,301	0,146	0,314	0,803	0,295	0,169	0,758	0,586	0,991	0,417	0,560
RMSSD	0,011	0,005	0,000	0,416	0,000	0,000	0,045	0,250	0,349	0,159	0,296	0,283	0,336	0,854	0,350	0,346	0,827	0,961	0,450	0,451	0,529
BSTRi	0,916	0,000	0,012	0,031	0,004	0,045	0,000	0,023	0,009	0,000	0,000	0,000	0,000	0,002	0,000	0,065	0,003	0,173	0,313	0,001	0,004
Mean HR	0,003	0,000	0,852	0,000	0,063	0,250	0,023	0,000	0,000	0,041	0,039	0,061	0,036	0,125	0,036	0,283	0,122	0,435	0,588	0,062	0,141
Mean RR	0,001	0,000	0,840	0,000	0,029	0,349	0,009	0,000	0,000	0,038	0,037	0,037	0,027	0,058	0,026	0,148	0,036	0,182	0,562	0,038	0,080
Stand Up Time (fast)	0,182	0,002	0,478	0,047	0,171	0,159	0,000	0,041	0,038	0,000	0,000	0,000	0,000	0,000	0,000	0,001	0,000	0,149	0,324	0,000	0,000
Walk Away Time (fast)	0,150	0,007	0,682	0,041	0,301	0,296	0,000	0,039	0,037	0,000	0,000	0,000	0,000	0,000	0,000	0,001	0,000	0,283	0,063	0,000	0,000
Rotatiion time (fast)	0,262	0,008	0,496	0,067	0,146	0,283	0,000	0,061	0,037	0,000	0,000	0,000	0,000	0,000	0,000	0,000	0,000	0,106	0,146	0,000	0,000
Walk Back Time (fast)	0,094	0,006	0,829	0,037	0,314	0,336	0,000	0,036	0,027	0,000	0,000	0,000	0,000	0,000	0,000	0,001	0,000	0,222	0,061	0,000	0,000
Sit Down Time (fast)	0,105	0,024	0,985	0,111	0,803	0,854	0,002	0,125	0,058	0,000	0,000	0,000	0,000	0,000	0,000	0,008	0,000	0,827	0,273	0,001	0,003
Total Time (fast)	0,113	0,005	0,719	0,037	0,295	0,350	0,000	0,036	0,026	0,000	0,000	0,000	0,000	0,000	0,000	0,000	0,000	0,248	0,082	0,000	0,000
Rotation steps (fast)	0,443	0,091	0,667	0,298	0,169	0,346	0,065	0,283	0,148	0,001	0,001	0,000	0,001	0,008	0,000	0,000	0,020	0,233	0,099	0,001	0,001
Step correlation (fast)	0,061	0,026	0,790	0,116	0,758	0,827	0,003	0,122	0,036	0,000	0,000	0,000	0,000	0,000	0,000	0,020	0,000	0,062	0,477	0,000	0,000
Step frequency (fast)	0,302	0,223	0,884	0,425	0,586	0,961	0,173	0,435	0,182	0,149	0,283	0,106	0,222	0,827	0,248	0,233	0,062	0,000	0,886	0,252	0,493
Sway (fast)	0,451	0,805	0,572	0,613	0,991	0,450	0,313	0,588	0,562	0,324	0,063	0,146	0,061	0,273	0,082	0,099	0,477	0,886	0,000	0,030	0,010
Step number (fast)	0,049	0,025	0,740	0,062	0,417	0,451	0,001	0,062	0,038	0,000	0,000	0,000	0,000	0,001	0,000	0,001	0,000	0,252	0,030	0,000	0,000
Step length (fast)	0,084	0,057	0,735	0,138	0,560	0,529	0,004	0,141	0,080	0,000	0,000	0,000	0,000	0,003	0,000	0,001	0,000	0,493	0,010	0,000	0,000

Table S3. Statistical test p-values of correlations between clinical and demographical parameters and HRV variables.

	**Age**	**Disease duration**	**Hoehn & Yahr**	**MoCA**	**UPDRS III**	**LEDD**	**FOGQ**	**Stand Up Time**	**Walk Away Time**	**Rotation Time**	**Walk Back Time**	**Sit Down Time**	**Total Time**	**Rotation steps**	**Step correlation**	**Step frequency**	**Sway**	**Step number**	**Step lenght**
Age	0,000	0,223	0,874	0,126	0,115	0,991	0,373	0,288	0,149	0,251	0,116	0,166	0,137	0,217	0,272	0,206	0,048	0,070	0,024
Disease duration	0,223	0,000	0,085	0,626	0,066	0,034	0,682	0,192	0,139	0,104	0,111	0,268	0,121	0,045	0,391	0,180	0,025	0,109	0,147
Hoehn & Yahr	0,874	0,085	0,000	0,283	0,005	0,525	0,137	0,119	0,088	0,094	0,075	0,332	0,088	0,025	0,040	0,033	0,266	0,016	0,020
MoCA	0,126	0,626	0,283	0,000	0,089	0,419	0,327	0,003	0,001	0,038	0,000	0,009	0,001	0,259	0,006	0,932	0,153	0,001	0,003
UPDRS III	0,115	0,066	0,005	0,089	0,000	0,528	0,069	0,001	0,000	0,002	0,000	0,003	0,000	0,026	0,013	0,096	0,055	0,000	0,000
LEDD	0,991	0,034	0,525	0,419	0,528	0,000	0,069	0,202	0,219	0,075	0,226	0,292	0,185	0,128	0,162	0,090	0,255	0,439	0,560
FOGQ	0,373	0,682	0,137	0,327	0,069	0,069	0,000	0,016	0,021	0,002	0,031	0,068	0,016	0,020	0,003	0,004	0,771	0,059	0,176
Stand Up Time	0,288	0,192	0,119	0,003	0,001	0,202	0,016	0,000	0,000	0,000	0,000	0,000	0,000	0,001	0,000	0,075	0,220	0,000	0,000
Walk Away Time	0,149	0,139	0,088	0,001	0,000	0,219	0,021	0,000	0,000	0,000	0,000	0,000	0,000	0,000	0,000	0,076	0,082	0,000	0,000
Rotation Time	0,251	0,104	0,094	0,038	0,002	0,075	0,002	0,000	0,000	0,000	0,000	0,000	0,000	0,000	0,000	0,048	0,160	0,000	0,000
Walk Back Time	0,116	0,111	0,075	0,000	0,000	0,226	0,031	0,000	0,000	0,000	0,000	0,000	0,000	0,000	0,000	0,068	0,072	0,000	0,000
Sit Down Time	0,166	0,268	0,332	0,009	0,003	0,292	0,068	0,000	0,000	0,000	0,000	0,000	0,000	0,001	0,000	0,360	0,101	0,001	0,002
Total Time	0,137	0,121	0,088	0,001	0,000	0,185	0,016	0,000	0,000	0,000	0,000	0,000	0,000	0,000	0,000	0,072	0,080	0,000	0,000
Rotation steps	0,217	0,045	0,025	0,259	0,026	0,128	0,020	0,001	0,000	0,000	0,000	0,001	0,000	0,000	0,005	0,161	0,085	0,000	0,000
Step correlation	0,272	0,391	0,040	0,006	0,013	0,162	0,003	0,000	0,000	0,000	0,000	0,000	0,000	0,005	0,000	0,032	0,312	0,000	0,000
Step frequency	0,206	0,180	0,033	0,932	0,096	0,090	0,004	0,075	0,076	0,048	0,068	0,360	0,072	0,161	0,032	0,000	0,679	0,068	0,152
Sway	0,048	0,025	0,266	0,153	0,055	0,255	0,771	0,220	0,082	0,160	0,072	0,101	0,080	0,085	0,312	0,679	0,000	0,036	0,016
Step number	0,070	0,109	0,016	0,001	0,000	0,439	0,059	0,000	0,000	0,000	0,000	0,001	0,000	0,000	0,000	0,068	0,036	0,000	0,000
Step lenght	0,024	0,147	0,020	0,003	0,000	0,560	0,176	0,000	0,000	0,000	0,000	0,002	0,000	0,000	0,000	0,152	0,016	0,000	0,000
